# Advancing reef recovery through insights into coral nutrition

**DOI:** 10.1016/j.isci.2026.114747

**Published:** 2026-01-20

**Authors:** Jennifer L. Matthews, Christine Ferrier-Pagès, Jonathan Moorhead, Raquel S. Peixoto, David Raubenheiemer, Liza M. Roger, David J. Suggett, Martin Tresguerres, Madeleine J.H. van Oppen, Christian R. Voolstra, Emma F. Camp

**Affiliations:** 1 Climate Change Cluster, University of Technology Sydney, Ultimo, NSW 2007, Australia; 2 Equipe Ecophysiologie Corallienne, Centre Scientifique de Monaco, Monaco, MC 98000, Monaco; 3 Monsoon Aquatics, Burnett Heads, QLD 4670, Australia; 4 Division of Biological and Environmental Science and Engineering (BESE), King Abdullah University of Science and Technology (KAUST), Thuwal, Saudi Arabia; 5 Charles Perkins Centre, The University of Sydney, Sydney, NSW 2006, Australia; 6 School of Life and Environmental Sciences, The University of Sydney, Sydney, NSW 2006, Australia; 7 Arizona State University, School of Molecular Sciences, 551 E University Dr, Tempe, AZ 85281, USA; 8 Arizona State University, School of Ocean Futures, 777 E University Dr, Tempe, AZ 85281, USA; 9 KAUST Coral Restoration Initiative (KCRI), King Abdullah University of Science and Technology (KAUST), Thuwal, Saudi Arabia; 10 Marine Biology Research Division, Scripps Institution of Oceanography, University of California, San Diego, La Jolla, CA 92093, USA; 11 School of Biosciences, The University of Melbourne, Parkville, VIC 3010, Australia; 12 Australian Institute of Marine Science, PMB 3, Townsville MC, Townsville, QLD 4810, Australia; 13 Department of Biology, University of Konstanz, Konstanz 78457, Germany

**Keywords:** Natural sciences, Environmental science, Nature conservation, Biological sciences

## Abstract

Coral reefs support immense biodiversity and human well-being, yet accelerating environmental change demands new strategies to strengthen reef resilience. Across ecological systems, balancing nutritional supply with organismal demand is fundamental to performance and persistence, but this principle has been rarely applied to corals. We propose a nutritional ecology framework that links coral physiological requirements with environmental nutrient supply and quality. By systematically addressing critical knowledge gaps of coral physiological requirements – including nutrient balance, quality, acquisition, and allocation – and integrating these with environmental nutrient data, a nutritional ecology framework can identify mismatches between nutritional requirements and availability that impact coral performance under stress. These insights provide a foundation for advancing restoration practices, from site selection to propagation effectiveness, while opening opportunities for interventions such as targeted nutrient supplementation or microbiome-based nutrient enhancement.

## Graphical abstract

**Figure:**
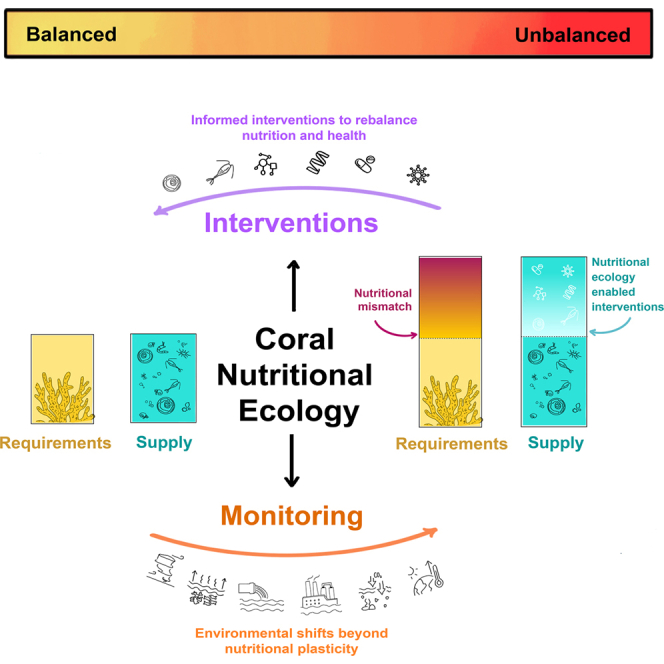

## Introduction

Coral reef ecosystems are under threat from marine heat waves,^1^ deoxygenation and ocean acidification, as well as local pressures such as eutrophication, pollution, and overfishing.^2^ The predicted global loss of coral reefs carries profound social, ecological, and economic repercussions.^3^^,^^4^ Consequently, enhanced protective strategies (e.g., the Kunming-Montreal Global Biodiversity Framework) and targeted funding opportunities (e.g., Global Fund for Coral Reefs, CORDAP) have already been initiated in response to the recent rapid decline of coral reefs worldwide. However, a persistent challenge for reef management is ensuring that corals - the foundation of reef ecosystems - can tolerate and recover from inevitable environmental change.^5^ While addressing the drivers of climate change remains essential, the slow pace of emissions reductions and delayed ocean recovery underscore the need for tailored management strategies that can rapidly enhance coral physiological resilience.^6^^,^^7^

One promising but underutilized avenue is nutrition (Box 1). Despite decades of fundamental research highlighting the importance of nutrition for coral fitness (e.g., Muscatine & Porter, 1977^25^), our understanding of optimal coral nutrition, particularly during periods of stress, remains unresolved. Consequently, nutritional considerations are rarely incorporated into conservation or restoration, even though in other fields, in agriculture, human health, and wildlife conservation, identifying and correcting nutritional imbalances have transformed outcomes. For example, identification of imbalances in amino acids, lipids, and micronutrients has enabled corrections to improve crop yields and pollinator health, and even save critically endangered birds.^21^^,^^26^ This gap suggests that while the importance of coral nutrition is well recognized, we lack the integrative framework needed to make coral nutrition actionable.

Box 1 **Key definitions**

**Nutrients** are chemical substances used by organisms to grow, reproduce, and survive. They can be metabolized to create energy or build cellular structures. **Macronutrients** are required in large quantities (e.g.,^8^ calcium,^9^ carbohydrates, protein, and lipids^8^). **Micronutrients** are needed in smaller amounts but play critical roles in biochemical processes (e.g., vitamins, trace elements such as selenium and iodine).

An essential nutrient is a nutrient required for physiological function that cannot be synthesized by the organism – either at all or in sufficient quantities – and essential nutrients are indispensable for various cellular metabolic processes and for the maintenance and function of the organism. Both macro and micronutrients can be **essential**. Some are **conditionally essential**,^10^ required only under particular conditions (e.g., manganese during heat stress in *Stylophora pistillata.*^11^).

**Nutrition** is the set of biochemical and physiological processes an organism uses to obtain and use nutrients.^12^ Autotrophs (e.g., plants and phytoplankton) synthesize most macronutrients with some exceptions (e.g., B vitamins^13^^,^^14^) and take up dissolved micronutrients, whereas heterotrophs acquire both through feeding and through translocation from microbial symbionts.

**Biogeochemical niche** (BN) refers to the chemical and biological conditions that define the nutrient space an organism occupies and interacts with.^15^ For corals, we expand this to include not only elements (e.g., C, N, and P) but also compounds (e.g., amino acids, fatty acids, or vitamins) exchanged among holobiont members.^16^^,^^17^ Approaches to characterizing an organism’s biogeochemical niche differ by scale. **Ecological stoichiometry** examines elemental ratios (e.g., C:N:P) and how they affect growth and nutrient cycling. **Ionomics** quantifies elemental concentration and links them to ecosystem-level processes.^18^^,^^19^ **Biochemical composition** analyses (e.g., lipids, proteins, carbohydrates, amino acids, and fatty acids) assess nutrient identity, energy content, and physiological pathways,^20^ leading to functions. Together, these methods can offer a multi-scale view of coral nutrition, helping to connect biochemical processes with ecological outcomes (Figure 3A).

**Nutritional landscape** is a conceptual and visual tool that represents how multiple nutrients are balanced to meet metabolic demands^17^ (Figure 3B). It highlights trade-offs, constraints, and interactions among nutrients that shape growth, reproduction, and resilience. For corals, the nutritional landscape describes the multidimensional space defined by nutrient availability, intake, and balance, and whether environmental profiles align - or not - with the organism’s nutritional targets. Mismatches in this space can signal reduced performance or resilience.

**Nutritional ecology** is an interdisciplinary field that examines how nutrition links organisms with their environments.^18^^,^^21^^,^^22^^,^^23^ It integrates four key concepts: (1) environmental conditions and nutrient availability, (2) organism requirements, (3) nutrient acquisition strategies, and (4) nutrient allocation (Figure 3C). Applied to corals, this framework can explain how nutrition shapes performance and resilience, and how mismatches between demand and supply arise. For instance, in wild bees, nutritional ecology has been used to link floral nutrient composition to bee health and population dynamics.^24^

**Figure 1:**
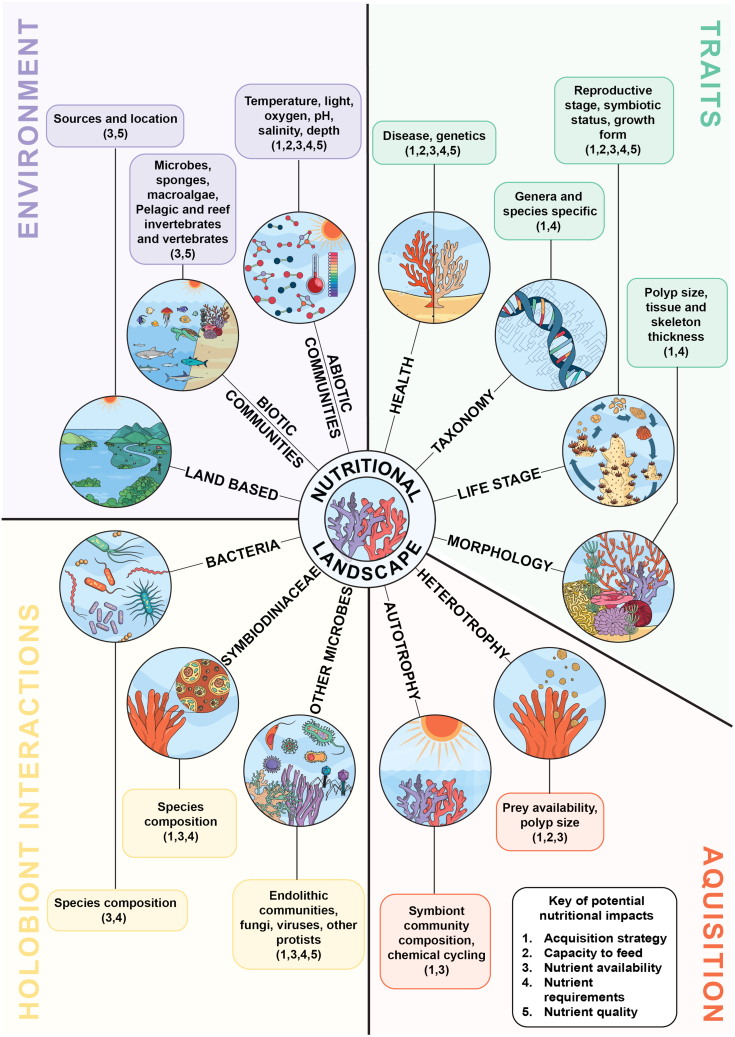
Key factors influencing coral nutrition Twelve central factors are grouped into four overarching themes, each shaping coral nutrition. For each factor, example considerations are represented in colored boxes, with potential nutritional impacts numbered according to the key. Further details and discussion are provided in the main text.

Corals are holobionts, consisting of the coral hosts and their diverse microbial partners, including Symbiodiniaceae microalgae and other microeukaryotes, archaea, bacteria, viruses.^27^^,^^28^ This integrated metabolic system^29^ shapes both nutrient utilization and availability at colony and reef scales.^30^^,^^31^^,^^32^^,^^33^ Coral nutrition is further influenced by biotic interactions (e.g., microbes, grazers, and neighbors), abiotic factors (e.g., light, temperature, and nutrient availability), and intrinsic traits (e.g., polyp size and modes of nutrient acquisition) (Figure 1). Thus, coral health is fundamentally linked to nutrition - the outcome of interactions between holobiont traits and environmental conditions, including nutrient supply.

**Figure 2:**
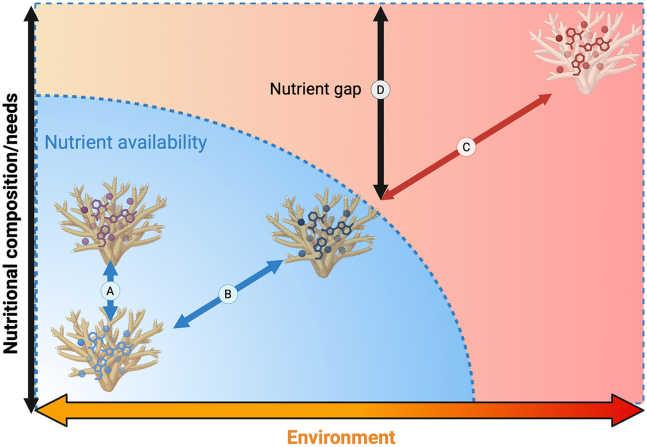
Coral nutritional plasticity Corals can have nutritional flexibility within their physiological and available nutritional range (blue area) based on (A) biotic and (B) abiotic conditions. However, the chemical space within which corals can persist may not allow for future environmental changes (C; red area). The difference is a nutritional mismatch that needs to be filled for corals to survive (D). Created in BioRender (https://BioRender.com/zbvgcfx).

At the heart of coral holobiont function is nutrient balance - the alignment between nutrient intake and the metabolic demand. Disruptions to this balance are the hallmark of coral stress^34^ cascading from individual corals to entire reef ecosystems. Environmental change disrupts balance by altering nutrient availability,^35^^,^^36^ modifying coral nutritional demands,^37^^,^^38^ and affecting nutrient quality - both identity (element, macromolecule, or compound) and value (bioavailability and functional utility).^20^ Corals can be nutritionally flexible and persist under conditions that appear suboptimal, but relative mismatches - the gap between environmental supply and physiological requirements^18^ - can still limit performance, particularly under other stressors.^34^ Severe imbalances can push corals outside their functional chemical space, resulting in mortality if not corrected^15^^,^^20^ (Figure 2). Thus, balance - not just availability - operates as the critical target for understanding and managing coral nutrition.

**Figure 3:**
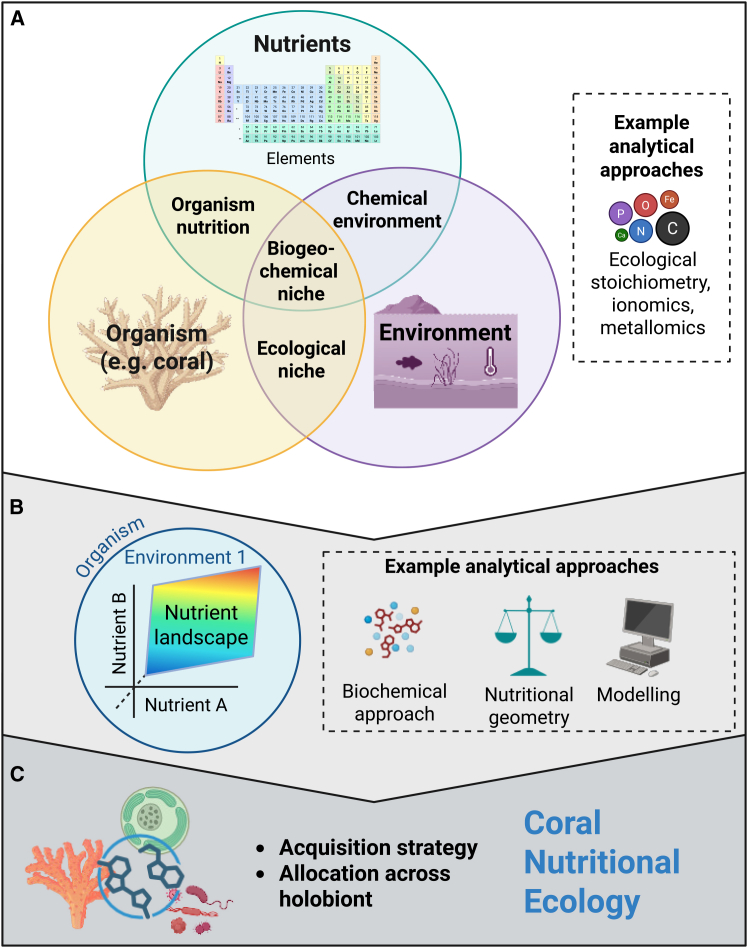
Nutritional ecology framework for corals (A) The biogeochemical niche describes both the environmental availability of elements (e.g., C, N, and P) and the coral’s requirements, including the quality of nutrients (identity and bioavailability), depending on the organism (including the evolutionary state of an individual and innate factors contributing to homeostasis) and its abiotic and biotic environment. Nutrient composition can be quantified using complementary approaches: ecological stoichiometry (elemental ratios), ionomics (elemental concentrations), and metallomics (trace metal concentrations). (B) Nutritional geometry (an analytical framework for visualizing multidimensional trade-offs) and modeling analysis of the coral macromolecules (e.g., proteins, lipids, and carbohydrates) can map a coral’s nutritional landscape - a multidimensional representation of how nutrient intake aligns with metabolic demand, and where trade-offs or constraints emerge. (C) Coral strategies for nutrient acquisition and allocation within the holobiont further shape how nutrition influences performance and resilience under changing environments. When combined with organismal requirements and environmental composition, these perspectives form an integrated nutritional ecology framework for corals. Created in BioRender (https://BioRender.com/e6tvr88).

To study nutrient balance, we need tools that link physiology with the environment (Box 1). As with all organisms, corals occupy a biogeochemical niche (BN) – the range of environmental chemical conditions that provide the bioelements they use, modify, or depend on^15^ (Figure 3). Understanding an organism’s BN can reveal its functional role within an ecosystem^39^ and predict responses to environmental change. Ecological stoichiometry (ES) provides a useful starting point for characterizing the BN of a species by examining elemental ratios (e.g., C:N:P).^40^ However, its predictive power is limited in complex systems such as coral reefs, where corals can compensate for imbalances by consuming larger amounts of low-quality nutrients. For example, plankton rich in essential amino acids are high quality, while detrital carbon, although abundant, is of low quality.^41^^,^^42^

To make BN approaches more relevant, ES must be combined with measures of nutrient quality (identity and value), acquisition strategies, and allocation. Analytical approaches such as biochemical assays (lipid, protein, and carbohydrate composition) and stable isotope analyses can help resolve nutrient identity, value, and flow through the holobiont. By linking these data to environmental variables, researchers can map the coral nutritional landscape – a multidimensional view of how well the coral diet fulfills their needs under different conditions (Figure 3). Nutritional landscapes highlight balance, trade-offs, and constraints, but they represent the outcome rather than the mechanism. They do not capture key physiological processes such as acquisition strategies or nutrient allocation within the holobiont.

This is where nutritional ecology – shown to improve management in other ecosystems^18^^,^^43^ – provides the next step. By linking the environmental availability of nutrients with coral nutritional balance, nutrient quality, acquisition strategies, and allocation patterns, nutritional ecology provides an integrated framework to explain and predict how nutrition shapes coral performance and ecological roles.^20^ More importantly, nutritional ecology allows researchers to identify nutritional mismatches, which then provides clear intervention points: by knowing when and where supply fails to meet demand, managers and researchers can design targeted strategies – from site selection and restoration design, to developing nutritional supplements – that directly support coral survival under stress.^20^ We also highlight emerging technologies and intervention strategies that can be applied or improved to manage nutrition at the coral scale as a first step toward broader conservation outcomes.

## Drivers of nutritional mismatch in corals

In the Anthropocene, several stress factors alter the nutritional stability of corals. Climate drivers such as ocean warming, CO_2_-induced nutrient dilution,^44^ and deoxygenation,^35^^,^^36^ interact with local pressures, including land run-off^45^ to disrupt coral nutrition. This disruption occurs both by altering coral physiology and by modifying the availability, distribution, and quality of nutrients^,^.^15^^,^^44^^,^^46^^,^^47^

Corals typically function within a nutrient ratio that reflects their biogeochemical niche.^48^^,^^49^^,^^50^ Yet environmental change shifts nutrient requirements by altering biotic interactions,^51^ holobiont composition,^29^^,^^52^^,^^53^ and metabolic rates.^54^ For example, when temperatures exceed tolerance limits, loss of Symbiodiniaceae (coral bleaching) reduces autotrophic input^45^ and forces corals to adjust nutrient acquisition strategies.^55^^,^^56^^,^^57^^,^^58^^,^^59^ Responses are context dependent^60^ some species increase heterotrophy,^61^ although this is energetically costly and constrained by the availability or low quality of available food. Similarly, rising CO_2_ may boost inorganic carbon for photosynthesis but often dilutes essential nutrients such as trace elements, lowering nutrient quality in oligotrophic systems.^44^

Nutrient mismatches impact holobiont function,^34^^,^^62^^,^^63^ reduce coral resilience, and increase susceptibility to disease and mortality.^64^^,^^65^ At ecosystem scales, sustained nutritional disruption accelerates coral loss, drives algal phase shifts,^66^ promotes microbialization,^67^ and alters dissolved organic carbon cycling - changes that reverberate across reef food webs.^18^^,^^45^^,^^53^^,^^68^^,^^69^

Despite the clear links between coral nutrition, stress, and resilience, we still lack a predictive understanding of which nutrients are most important and when, how they are acquired and allocated, and how these processes vary across taxa and environments (Table 1). Addressing these gaps is essential if nutritional ecology is to evolve from a concept to supporting targeted and actionable coral conservation strategies. Later in discussion, we discuss in more detail some of the key knowledge gaps.

**Table 1:** Summary of the current knowledge and gaps in coral nutrition

	Gap in knowledge	Relevance	Knowns	Unknowns	Approaches
1. Nutrient requirements and quality	Minimum macro- and micronutrient requirements of corals	Critical for defining baseline nutrient needs	Corals require elements such as C, N, P, Ca, Mg, and trace elements (e.g., Fe, Zn, Mn, and Cu) for metabolism, photosynthesis, and stress response	Exact quantitative requirements across species, life stages, and environmental conditions	Elemental analysis, physiological assays across species and life stages, targeted nutrient addition studies Scales: holobiont, species
	Influence of environmental variability on holobiont nutrient dynamics	Informs coral resilience and reef management under climate change	Temperature, pH, and nutrient changes alter metabolic demands and nutrient flows	Interactive effects of multiple stressors: long-term adaptability of nutrient cycling	Environmental manipulations (multi-stressor experiments), multi-omics, physiological monitoring (longitudinal omics profiling) Scales: holobiont, ecosystem
	Impact of trace metal stoichiometry and macromolecule ratios	Critical for water quality management and coral supplementation strategies	Trace metals such as Fe, Zn, Mn are essential; some stoichiometric ratios (e.g., C:N:P) are studied	Roles of C:metal or compound-level stoichiometry; thresholds for deficiency/toxicity	Elemental profiling under controlled gradients, stoichiometric modeling Scales: holobiont, ecosystem
	Role of specific compounds in coral nutrition	Identifies high-value nutrient targets for boosting coral resilience	Some compounds (e.g., glucose and docosahexaenoic acid) have known roles in symbiosis and stress response; metabolomics/lipidomics reveal chemical diversity	Full identification and function of compound classes; nutritional roles of unidentified metabolites	Metabolomics, lipidomics, compound-specific isotope analysis, time-series, and under variable conditions, cross-species compound mapping Scales: cellular, holobiont
2. Mode of nutrient acquisition	Trophic plasticity across coral species and conditions	Key to understanding adaptability in changing environments	Corals can shift between autotrophy and heterotrophy based on conditions; some use DOM/POM/zooplankton	Extent of plasticity across species; thresholds for shifts; long-term fitness outcomes	Bulk stable isotope analysis, compound-specific isotope analysis, longitudinal feeding studies, metabolomics, and environmental manipulations Scales: holobiont, species
	Nutrient acquisition rates and digestion/assimilation mechanisms	Helps optimize coral feeding strategies for restoration or intervention	Corals use nematocysts, cilia, and mucus nets for capture; early studies on prey selection exist	Species-specific assimilation efficiencies; digestion under stress; prey-to-biomass conversion rates	Feeding trials, microscopy, gut content analysis, isotope-labelled prey studies, and digestive enzyme assays Scale: organism
	Coral feeding strategies on pico- and nanoplankton	Improves understanding of nutrient dynamics in oligotrophic environments	Feeding on small plankton contributes to nutrition; some morphological adaptations are known	Extent, efficiency, and role of this feeding mode across coral taxa and environments	Feeding trials, microscopy, flow cytometry, prey uptake quantification, particle tracer experiments Scales: organism, holobiont
3. Nutrient allocation	Nutrient allocation among holobiont members and compartments	Enables targeted interventions and understanding of stress resilience	Isotope tracing has shown nutrient partitioning; nutrient reallocation during stress is documented	Precise allocation dynamics under different stressors; cell-specific nutrient demand timing	Stable isotope tracers, mass spectral imaging, cell-specific tracer tracking Scales: cellular, holobiont

## Knowledge gap 1: Nutrient requirements and quality

Corals require a specific blend of nutrients to ensure their survival. These requirements vary with environment, life stage, taxonomy, phenotype, and symbiotic state^55^^,^^60^^,^^70^^,^^71^^,^^72^^,^^73^^,^^74^^,^^75^^,^^76^ (Figure 1). Yet despite decades of research, we still lack a quantitative understanding of what constitutes the minimum and the optimal nutrition for different coral species and contexts. For example, scleractinian corals generally consume more dissolved inorganic nutrients than octocorals,^60^ while some taxa efficiently use dissolved organic matter, and others prefer particulate organic matter (POM) or specific zooplankton.^77^ Such diversity underscores that the nutrient requirements of corals are not uniform across corals but depend on a combination of intrinsic traits and external conditions. Importantly, responses to nutrient supply can vary not only among species but also among individuals within a species,^78^^,^^79^ underscoring the need to incorporate intraspecific diversity when establishing nutritional baselines.

Essential elements such as carbon, nitrogen, phosphorus, calcium, and magnesium^69^ are known to underpin coral health,^68^^,^^69^^,^^80^^,^^81^ and trace elements such as iron, manganese and zinc, have recognized roles in photosynthesis and enzyme function.^82^ However, the concentrations required, the thresholds for deficiency or toxicity, and the importance of less-studied ratios (e.g., C:trace metals^50^^,^^83^^,^^84^^,^^85^) are not well understood.^85^ Furthermore, the precise mode of action remains unresolved. For example, manganese is implicated in photosynthesis and antioxidant defense, but the specific molecules and pathways that require Mn remain unclear.^85^ Optimizing trace metal availability is already being explored for corals^86^ making it imperative to close these knowledge gaps to expedite learnings and support informed decision-making.

Similarly, while the importance of proteins, lipids, and carbohydrates is established,^16^^,^^87^^,^^88^ few studies have resolved their roles at the level of specific compounds.^89^ For example, polyunsaturated fatty acids, such as docosahexaenoic acid (DHA), can enhance thermal tolerance^45^^,^^90^ yet baseline concentrations across coral taxa are unknown. Considering such compounds highlights the need to consider coral nutrition in a multidimensional context, rather than as single currencies such as carbon content.^23^ Fluctuating abiotic factors (temperature, light, pH) and biotic changes (e.g., shifts in symbiont identity or macroalgal overgrowth) further reshape the nutritional landscape on daily to seasonal scales, creating feedback loops that alter both nutrient availability and demand,^45^^,^^81^^,^^91^^,^^92^ ultimately affecting coral health.^93^

**Figure 4:**
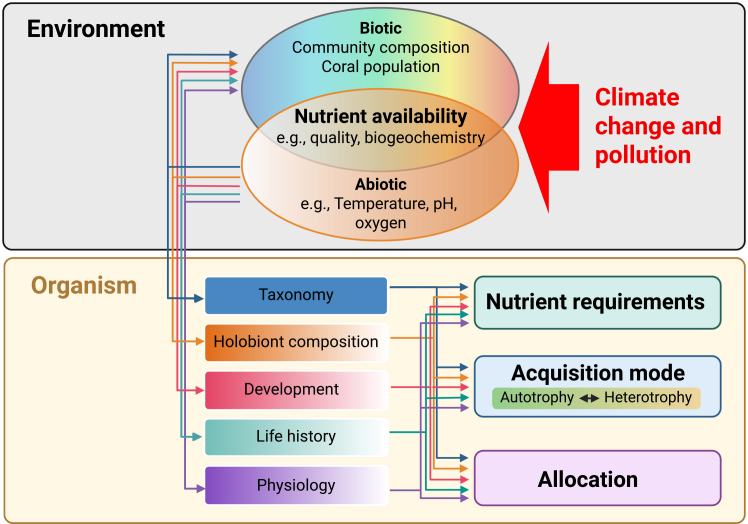
The core research components of coral nutritional ecology The core components of coral nutritional ecology are the environment and the organism. The environment is partitioned into biotic and abiotic components. These can drive coral phenotypic variables, including holobiont composition, development, including morphology, life history, current physiology, alongside static factors such as coral taxonomy. These organismal traits then define the nutrient requirements (including quality), acquisition mode, and allocation. The arrows connecting environment and organisms indicate the bidirectional interactions that take place between coral nutritional requirements and the environment; for example, the effects of the environment on the phenotype (downward arrows) and the impact of the phenotype on the biotic environment (upward arrows). A nutritional ecology framework should also consider the interaction between biotic and abiotic environments, and with the intention of informing restoration, alongside perturbations.^20^ This is represented by the red arrow from climate change and pollution affecting the environment, thus subsequently influencing coral organism nutrition. Created in BioRender (https://BioRender.com/a0ry1ms).

Equally critical is the lack of knowledge concerning nutrient availability and turnover in reef environments. Although shallow waters are often assumed to be nutrient poor, studies (e.g., Red Sea^94^) show that corals such as *Stylophora* maintain similar heterotrophic intake across depths, challenging this view. Resolving such uncertainties requires high-resolution profiles of plankton, DOM, POM, and micronutrients, alongside measurements of microbial recycling. The “extended microbial loop” – from bacterial uptake through phytoplankton growth to zooplankton grazing – remains poorly quantified, and apparent zooplankton scarcity may simply reflect rapid production balanced by rapid consumption. Without such baselines, it is impossible to match coral nutritional requirements with actual supply, and few studies currently attempt to integrate these dynamics within a holistic framework of environmental supply and holobiont biology (Figure 4).

**Figure 5:**
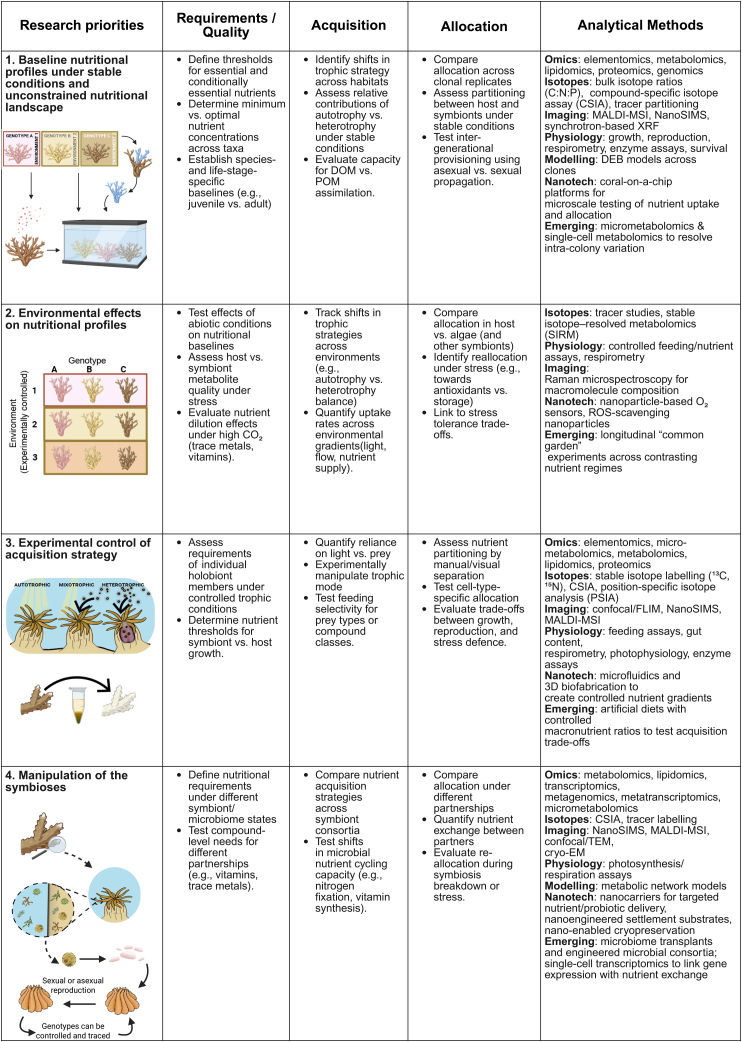
Pathway to filling the knowledge gaps required to apply a nutritional ecology framework This table outlines experimental approaches to bridge critical knowledge gaps in coral nutrition, linking requirements, acquisition, and allocation with analytical methods. Traditional tools (e.g., stoichiometry, isotopes, and physiology) are complemented by emerging techniques such as micrometabolomics, synchrotron-based imaging, artificial diets, and microbiome transplants, as well as nanotechnologies for nutrient sensing and delivery. These approaches provide a roadmap for building the information required for a nutritional ecology framework that integrates coral physiological requirements with environmental nutrient data, enabling the identification of mismatches and the development of targeted interventions. Created in BioRender (https://BioRender.com/3nvd71a).

In short, we know corals require a diverse suite of elements and compounds, but we lack quantitative thresholds, compound-level resolution, a mechanistic understanding of nutrient mode of action, and a multidimensional framework linking requirements with environmental variability. Closing this gap through experimental evidence and computational modeling with physiological and environmental data (Figure 5) is critical for predicting resilience and designing targeted nutritional strategies for coral conservation and restoration. Elementomics – the study of the elemental composition (abundance and stoichiometry) – reveals changes in bioelement production under varied external stimuli and determines which nutrients are necessary for growth.^95^ Metabolomics and lipidomics now allow simultaneous profiling of hundreds, and even thousands, of compounds, offering unprecedented insight into which metabolites are exchanged, depleted, or accumulated under stress.^95^ These approaches can help define nutrient requirements by identifying which elements and compounds are consistently essential across species and life stages, and which are conditionally important under stress (e.g., heat-induced demand for antioxidants or membrane lipids).^53^^,^^96^^,^^97^ They also refine the concept of nutrient quality by distinguishing specific elements or compounds (e.g., essential amino acids, polyunsaturated fatty acids, and vitamins) that are most valuable to coral metabolism and resilience. Applied across taxa and environments, metabolomics therefore provides both the baseline “nutritional fingerprint” of healthy corals and the compound-level markers of mismatch that signal vulnerability or adaptive capacity. Yet these approaches remain applied to only a handful of taxa and conditions. Without broader baselines, we cannot identify which compounds are universally essential, conditionally essential, or most useful as intervention targets. Furthermore, when integrated with isotopes (elemental or compound), the metabolic pathway can be resolved, shedding light on the value and optimum concentrations required. Defining nutrient requirements is not simply descriptive; it establishes the baseline against which environmental supply can be compared. Once these thresholds are known, they can be integrated into the nutritional ecology framework to identify mismatches and design targeted interventions.

## Knowledge gap 2: Mode of nutrient acquisition

Photosymbiotic corals thrive in oligotrophic reefs through mixotrophy, combining autotrophic inputs from Symbiodiniaceae, nutrient cycling with other holobiont partners, and heterotrophic feeding. Acquisition strategies shift along a continuum depending on environmental conditions,^53^^,^^55^^,^^98^ species,^99^ life stage, and health status, with this trophic plasticity appearing central to coral survival in marginal environments.^100^ Yet the limits and efficiency of this plasticity remain poorly understood.

Corals capture a wide range of nutrient particles^25^^,^^81^- plankton, DOM, and POM -^101^ using nematocysts, mucus nets, and other specialized structures.^25^^,^^64^^,^^65^^,^^66^^,^^67^^,^^68^^,^^69^^,^^81^^,^^101^^,^^102^^,^^103^ Feeding on pico- and nanoplankton is increasingly recognized as a significant source of nutrition,^45^^,^^70^^,^^104^ but rates of assimilation, digestion, and conversion to biomass remain largely unresolved.^100^^,^^105^ Feeding efficiency varies with prey availability, polyp morphology, life stage, and health status.^31^^,^^74^ While some feed opportunistically, others specialise,^77^^,^^102^ even consuming unusually large prey.^103^ However, we still know little about assimilation efficiency, digestion,^105^ and conversion of prey into biomass,^100^ limiting our ability to assess the energetic value of heterotrophy under stress.

Within the holobiont, nutrient acquisition and recycling between the partners (e.g., coral-Symbiodiniaceae-bacteria^97^^,^^106^^,^^107^^,^^108^) support growth and reef accretion,^81^ yet the dynamics of these exchanges – especially those involving lesser-known symbionts (e.g., fungi,^109^ protozoa,^110^ and viruses^111^) – remain largely unexplored.^29^ Advances in techniques such as metabolomics, lipidomics, and compound-specific isotope analysis have begun to map these interactions,^90^^,^^112^^,^^113^^,^^114^^,^^115^ but many metabolites are still unidentified,^97^^,^^116^ and the temporal and spatial dynamics of exchange are rarely captured.^94^^,^^117^ Most studies provide static snapshots,^118^ when in reality acquisition is highly dynamic across time, life stage, and even within different regions of a single colony.^51^^,^^119^ These gaps hinder efforts to link trophic plasticity with resilience. For example, some species can increase heterotrophy to offset bleaching-related carbon loss,^61^ but this strategy can be energy-costly and is constrained when available nutrients are scarce or of low quality. Without a quantitative understanding of the capacity and limits of trophic flexibility, we cannot predict which species are most vulnerable, nor design interventions to address them.

Filling the knowledge gaps of coral nutrient acquisition will require combining emerging tools. High-resolution isotope tracers can track the assimilation of specific nutrients (e.g., labeled amino acids or fatty acids) from capture to incorporation into host or symbiont biomass. Coupled with mass spectral imaging tools such as NanoSIMS or MALDI-MSI, these tracers can localise where in the tissue nutrients are digested and transferred. Emerging techniques such as longitudinal metabolomics and micrometabolomics can then reveal how acquisition strategies shift under stress (e.g., daily changes in uptake of dissolved organic matter versus plankton).^55^^,^^94^^,^^113^^,^^117^^,^^120^^,^^121^ These approaches make it possible to quantify not only what corals consume, but how nutrients are digested, assimilated, and exchanged within the holobiont. Nanotechnology techniques such as 3D fabrication could allow for the creation of controlled nutrient gradients to experimentally manipulate trophic mode.^122^ This shift from descriptive to mechanistic understanding is essential if nutrient acquisition strategies are to be leveraged in active reef management – for example, by identifying species with higher trophic plasticity for restoration. By incorporating environmental variables, we can further understand how environmental change shifts acquisition strategies, which allows us to predict the limits of trophic flexibility.^40^ Linking these shifts to nutrient requirements provides the foundation for experimental interventions, such as adjusting feeding regimes or supplementing limiting nutrients.^11^^,^^123^

## Knowledge gap 3: Nutrient allocation across cell types and holobiont members

Once nutrients are acquired, their allocation across coral hosts, symbionts, and microbial partners determines how effectively the holobiont functions. Corals can actively reallocate nutrients in response to stress, life stage, or genotype, prioritizing survival over growth and reproduction.^50^^,^^71^^,^^84^ For example, under heat stress or nutrient scarcity, resources may be diverted toward protective mechanisms such as antioxidant production,^124^^,^^125^ while growth and reproduction are reduced. Similarly, metals are regulated and redistributed according to physiological demands.^126^

Methods such as stable isotope tracer analysis and imaging mass spectrometry (e.g., NanoSIMS) have revealed partitioning patterns between autotrophic versus heterotrophic inputs and how these shift under environmental change.^55^^,^^113^^,^^120^^,^^121^^,^^127^ These studies suggest that when nutrient balance is disrupted, corals and their symbionts may redirect nutrients toward maintaining basic cellular function at the expense of nutritional exchange. Such reallocations can destabilize the coral-Symbiodiniaceae symbiosis, with downstream impacts on reproduction, immunity, and growth.^65^^,^^121^^,^^128^

Despite these advances, we still lack a mechanistic understanding of allocation rules: which nutrients are prioritized under stress, how allocation varies across species and life stages, and how microbial partners mediate redistribution. Without this knowledge, it is difficult to predict resilience or design interventions. Targeted elemental and metabolomic tracer studies with imaging, micrometabolomics, nanotechnology (e.g., targeted nanocarriers) techniques, and applications of microbial consortia engineering to diverse coral taxa could help resolve these dynamics and identify leverage points for nutritional interventions.^55^ For example, elemental stable isotope tracers (^13^C, ^1^N, ^3^S) or compound specific isotopes (e.g., glucose-13C_6_) can be pulsed into coral diets or dissolved nutrients, then tracked through host tissues and symbionts to quantify assimilation rates and nutrient allocation between partners. Coupled with high-resolution tools such as NanoSIMS or MALDI-imaging, these tracers can map nutrient flows at cellular or even subcellular scales, revealing which compounds are retained, recycled, or diverted under stress. Metabolomics (bulk and microscale) profiling then complements this by identifying shifts in key metabolites (e.g., amino acids, fatty acids, and vitamins) and linking them to environmental drivers. Applied to isolated tissues or cells (e.g., through “cell on a chip” nanotechnology^122^), these approaches can disentangle host-versus symbiont-specific nutrient use, providing a mechanistic view of holobiont partitioning. Proteomic analysis of transporters and enzymatic assays can provide more mechanistic evidence of the exchange. Performed in both controlled and field settings, researchers can determine not only what nutrients are limiting, but also when, where, and for whom within the holobiont they become critical. Resolving allocation dynamics moves us from knowing what corals acquire to understanding how nutrients are prioritized under stress. This mechanistic knowledge is essential for identifying leverage points where nutritional interventions can support resilience.

## Solutions to advance knowledge on coral nutrition: Applying nutritional ecology to the coral holobiont

Viewing coral reef ecology through the lens of nutrients and nutritional balance is not new – decades of research have examined how nutrient availability, quality, and cycling influence coral health (e.g., Muscatine and Porter 1977^25^). What is missing is an integrated framework that links this knowledge to actionable strategies for reef conservation and restoration. We argue that nutritional ecology provides such a framework by combining information on physiological requirements, environmental supply, nutrient acquisition, quality, and allocation within the holobiont.

Within this framework, addressing the knowledge gaps outlined is a prerequisite for action. Once nutrient requirements are quantified across species, life stages, and symbiotic states, these can be integrated with data on nutrient availability and quality across reef habitats. Experimental tests of how changes in nutrient profiles influence coral performance – both under controlled and field conditions – can then link physiology with environment. Addressing these knowledge gaps creates a roadmap for the application of nutritional ecology: from identifying needs, to mapping supply, predicting mismatches, forecasting ecological consequences, and finally designing interventions (Figure 2). Similar roadmaps in other fields show the value of this approach: nutritional ecology has improved wild bee health by optimizing floral nutrient profiles,^21^ and enhanced survival of endangered kākāpō by tailoring life-stage-specific supplements.^26^ Applying similar principles to corals could transform restoration by pinpointing when and where nutritional interventions are most effective.

To bridge the knowledge gaps and apply a nutritional ecology framework to corals, we propose four research priorities. First, characterize baseline nutritional requirements under stable conditions to establish the reference point for an unconstrained nutritional landscape. Second, assess how environmental changes (including the quality and availability of nutrients in the environment) alter nutritional profiles within and across coral species, linking physiological demand to ecological context. Third, examine how corals shift acquisition and allocation strategies under stress to reveal the limits of trophic plasticity and the trade-offs involved. And forth, investigate how microbial partners mediate nutrition, using new tools such as integrated omics^107^ and nanoscale imaging^107^^,^^129^ to capture exchanges at cellular and holobiont scales. Together, these priorities create the mechanistic foundation needed to integrate coral physiology (including nutrient requirements, acquisition, and allocation) with environmental nutrient data collected via elemental stoichiometry, ionomics, metallomics, biochemical profiling of plankton/DOM, and nutrient flux measurements in different reef habitats,^130^ allowing the nutritional ecology framework to move from concept to application (Figure 5).

Practical progress will depend on systematic experiments on carefully selected species, ideally those with well-documented nutrient acquisition modes, life cycles, and symbiotic diversity (e.g., Heit & Davy, 2024^91^) (Table S1). Such species provide tractable models to map detailed nutrient landscapes, while allowing broader comparative insights. Spatial heterogeneity within colonies should also be considered, as some genera host distinct micro-environments that affect nutrient flow.^40^^,^^131^ Furthermore, while studying individual species is immediately feasible, understanding the full diversity of coral nutritional strategies may take longer than they can endure, given the rapid pace of climate change.^132^ Computational modeling should therefore complement experiments, simulating coral metabolic pathways under different environmental scenarios; an approach successfully employed to inform wild pig conservation.^133^ Published genomic and metabolite data can serve as reference points, helping describe physiological responses under various environmental conditions and ensuring relevance across reef ecosystems. For corals, such models could predict which nutrients become limiting under future climates; this is the mismatch that nutritional intervention strategies can target.^56^ Such an approach balances experimental feasibility with the urgency of coral decline, moving nutritional ecology from concept to conservation tool.

## Solutions to address nutrient mismatch: Nutritional interventions to advance coral resilience and restoration

While challenging, rebalancing nutritional mismatches has been successfully achieved in other disciplines. In viticulture, correcting micronutrient deficiencies has improved crop yield and resilience.^134^^,^^135^ In marine ecology, supplementation strategies have supported the recovery of seabird and marine mammal populations.^88^^,^^136^ In human medicine, nutrient-based therapies are increasingly used to restore metabolic balance, such as in melanoma, where targeted nutrient delivery reactivated suppressed metabolic pathways.^137^ These examples illustrate a central principle: once nutritional mismatches are identified, they can be strategically corrected to improve resilience.

We contend that the same approach can be applied to corals. By using a nutritional ecology framework to identify when and where supply fails to meet demand, interventions can be designed to rebalance coral nutrition.^26^ Later in discussion, we outline four areas where this approach could be transformative.

### Strategic use of nutrition in coral propagation

Corals are increasingly propagated both *in situ* and ex situ for coral restoration purposes^138^ but nutritional considerations are rarely factored in. When corals are moved between environments, they are often exposed to nutrient regimes different from their original biogeochemical niche.^40^^,^^50^ For instance, corals collected from inner reefs may be propagated in mid-water nurseries (e.g., Howlett et al. 2023^139^), where they are exposed to different water flow, biotic communities, and nutrient profiles. Such mismatches could partially explain variable survival rates in outplanting projects.^138^ Accounting for these differences and selecting nursery sites or substrates with nutrient conditions closer to source reefs could improve outcomes. Ex situ culture also provides an opportunity to test nutritional tolerance ranges and acclimate corals to conditions beyond their natural niche before outplanting. Moreover, artificial structures could be engineered to leach or deliver beneficial compounds^140^ (e.g., embedding trace metals or calcium carbonate into cement that slowly dissolves, or using porous textures that trap plankton), providing a dual role as both substrate and nutrient source.

### Nutritional supplementation

Heterotrophic feeding can help corals compensate when symbiont productivity declines, but natural food sources are often scarce or of low quality.^131^ Nutritional supplementation – already standard practice in aquaculture – could therefore be harnessed in restoration. The process involves three steps: (i) identify limiting nutrients (e.g., phosphorus, trace metals, and essential fatty acids) through experiments, modeling, or using the trophic stoichiometric ratio (TSR) index^18^); (ii) design supplements that deliver these compounds in bioavailable forms; and (iii) select a delivery method suited to coral biology and site conditions. Live feeds such as rotifers and microalgae have long been used to rear corals *ex situ* and new technologies could support increased food availability in situ.^141^^,^^167^ Novel technologies offer new options, for example, biodegradable nanoparticles can encapsulate nutrients and release them gradually to coral tissues,^122^^,^^142^^,^^166^ while biocomposite films – thin, degradable polymer sheets that embed nutrients – can be placed near colonies to deliver compounds directly into the surrounding water, as demonstrated for curcumin.^123^^,^^142^ These approaches, already applied in biomedical science, could be adapted to reefs to support growth and reproductive output, stress tolerance, such as to sustain nutrition during bleaching, or improve post-outplant survival by allowing gradual *in situ* acclimation to new biochemical niches.

### Protection of areas identified as supporting nutrient balance under a climate change backdrop

Just as certain soils act as natural nutrient reservoirs in agriculture, some reef environments naturally provide more balanced nutrient conditions. Protecting these “nutrient refugia” within climate-ready marine protected areas^143^ could buffer corals from stress and enhance resilience. Identifying such sites requires a combination of tools: ecological stoichiometry to diagnose nutrient limitations (e.g., excess nitrogen relative to phosphorus favors algal growth over corals^45^), trace-metal profiling to reveal micronutrient status, and ecological surveys to link nutrient availability with coral health. Nutrient quotients (ratios such as N:P that are already used in soil restoration to indicate which nutrient is limiting^144^), could serve as simple diagnostic indicators of reef nutritional status. Machine learning and AI can also analyze large, multi-reef datasets to identify patterns in coral growth potential, complementing these diagnostic tools. Integrating these indicators into monitoring frameworks would help prioritize the protection of habitats most likely to sustain nutritional balance under climate change.

### Manipulate nutrient acquisition for holobiont members

The coral microbiome plays a pivotal role in nutrient cycling, from nitrogen fixation to vitamin synthesis.^144^^,^^145^^,^^146^^,^^147^^,^^148^^,^^149^ Harnessing these microbial functions could open a new frontier in nutritional interventions. Probiotics (direct addition of beneficial microbes), prebiotics (supplying compounds that favor beneficial microbes), and microbial metabolites^147^ could all be tailored and delivered to enhance coral nutrition^150^ – and/or their natural enhancement can be optimized through nutritional management. For example, enriching nitrogen-fixing bacteria could help buffer corals in nitrogen-limited environments,^148^ while providing targeted compounds might enhance photosynthetic efficiency or growth of Symbiodiniaceae.^107^^,^^151^^,^^152^ Early trials show promise in reducing bleaching and disease^112^^,^^153^^,^^154^^,^^155^^,^^156^ and the specific degradation of compounds (e.g., dimethylsulfoniopropionate) that may become toxic when overproduced during stress, but can serve as a nutrient if degraded by specific probiotics.^112^ Initial *in situ* risk assessments are underway and should be expanded to guide the safe and scalable advancement of this field.^157^^,^^158^^,^^159^ Analogous approaches in agriculture, such as fungal symbionts that boost plant nutrient acquisition, suggest untapped potential for coral restoration.^146^^,^^160^ Live feeds (e.g., microalgae) commonly utilized in the aquaculture industry could enhance nutrition while also delivering beneficial microorganisms for corals (BMCs).^146^

## Nutrition-based diagnostic tools

All of the above areas depend on accurate and timely diagnosis of nutritional stress. Biomarkers such as specific nutritional phenotypes such as heterotrophic compensation,^56^ oxidative stress enzymes,^161^ or microbial indicators of nitrogen limitation,^162^ could serve as early-warning tools.^168^ Metabolic biomarkers include enzymes such as superoxide dismutase and antioxidants such as glutathione, or metabolites such as inositol, all of which play significant roles in coral stress responses, including during suboptimal nutrition.^163^ Emerging technologies make this increasingly feasible: portable colorimetric or fluorometric assays for metabolic biomarkers, eDNA to track microbial shifts, cellular reactive oxygen species (ROS) sensors to measure stress response, and even chemical sensors (“e-noses”) capable of detecting volatile organic compounds as metabolic signatures,^95^ already being utilized for early warning systems for wildfires and harmful algal blooms. Coral e-noses could offer a non-invasive, cost-effective means of monitoring and protecting ecosystems, providing valuable data for informed decision-making in reef conservation and management efforts. These approaches could provide reef managers with rapid diagnostics to guide adaptive interventions, helping match the right supplement, microbial therapy, or propagation environment to the right corals.

## Conclusion: From coral to reef scales

The poor long-term prognosis for coral reefs creates an urgent need to best manage existing reefs while identifying new strategies to boost coral resilience. Undoubtedly, this requires immediate and fast-tracked efforts to reduce greenhouse emissions.^164^ However, the uncertain future for reefs requires concerted efforts to boost reef resilience through enhanced reef protection and the optimization of restoration techniques.^56^^,^^165^ Restoration needs augmentation to maximize outcomes, and here we have highlighted how a nutritional ecology approach can help to embed restoration within a scientific framework that has expedited learnings and outcomes in other ecosystems.^20^ The initial efforts to apply nutritional ecology principles should focus at the coral colony scale and then extend to the reef-scale by applying ionomics,^25^ ecological stoichiometry, and energy and nutrient flux frameworks.^130^ We acknowledge that significant effort will be required to translate some of the proposed research and development into scalable *in situ* solutions, and careful consideration of associated risks will be necessary.^143^ However, we propose that the risk of losing reefs is too great to allow these challenges to limit our efforts. Drawing on successes in agriculture, human health, and wildlife conservation, we provide a roadmap to employing a unifying framework of nutritional ecology, helping to move from fragmented insights toward actionable solutions to support coral persistence in a rapidly changing ocean.

## Acknowledgments

JLM was supported by a 10.13039/501100001775University of Technology Sydney Chancellor’s Research Fellowship and Pure Ocean Innovation award. RSP was supported by 10.13039/501100004052KAUST grant number BAS/1/1095-01-01. LMR was supported by NSF IntBio award 2316389. MT was partially supported by Revive & Restore's Advance Coral Toolkit. CFP was funded by the Government of the Principality of Monaco. We wish to acknowledge the Coral Research and Development Accelerator Platform (CORDAP) for their support to JLM and EFC to explore coral nutrition. We would like to acknowledge Maddison Shiels and Ellen Moynihan for their illustrative contributions to the graphical abstract and Figure 1.

## Author contributions

Conceptualization, J.L.M., C.F.P, and E.F.C.; writing – original draft, J.L.M.; and writing – review and editing – all authors.

## Declaration of interests

The authors declare no competing interests.
