# Supplementary material for: Advancing reef recovery through insights into coral nutrition
Source: iScience. 2026 Jan 20;29(2):114747. doi: 10.1016/j.isci.2026.114747 (PMC12886527; doi:10.1016/j.isci.2026.114747)
Supplement: Document S1. Table S1 [file mmc1.pdf]

iScience, Volume *29*

Supplemental information

Advancing reef recovery

through insights into coral nutrition

Jennifer L. Matthews, Christine Ferrier-Pagès, Jonathan Moorhead, Raquel S. Peixoto, David Raubenheiemer, Liza M. Roger, David J. Suggett, Martin Tresguerres, Madeleine J.H. van Oppen, Christian R. Voolstra, and Emma F. Camp

Supplemental Information

for

Advancing Reef Recovery Through Insights into Coral Nutrition

Jennifer L. Matthews[1*$], Christine Ferrier-Pagès[2], Jonathan Moorhead[3], Raquel S Peixoto[4], David Raubenheimer[5,6], Liza M. Roger[7,8], David J. Suggett[1,4,9], Martin Tresguerres[10], Madeleine J. H. van Oppen[11,12], Christian R Voolstra[13], Emma F. Camp[1*]

Supplemental Table 1. Examples of potential coral genera candidates to study nutrition. Numbers relate to the experimental opportunities described in Figure 5.

Coral genera	Pros (including model coral selection criteria)	Cons (including selection criteria)	Other considerations
Acropora	Geographically and environmentally widespread, with DNA extraction protocols and species genomes (1)<br><br>Mixotrophic. Acquisition strategy can be identified and controlled (3)<br><br>Easy to asexually propagate (1,4)<br><br>Multiple studies and examples of coral/Symbiodiniaceae separation (1-4)<br><br>Broadcast spawner, including ex situ, with aposymbiotic larvae (1,4)<br><br>Symbiodiniaceae and bacteria compositions described (1-4)	Difficult to rear in aquaria (3)	Can be chemically rendered Symbiodiniaceae-free (6)?<br><br>Metabolomics profiles under ranging conditions already exist.
Pocillopora	Geographically and environmentally widespread, including in extreme environments (e.g., mangroves). Species genomes exist (1). Mixotrophic (3)<br><br>Easy to maintain in aquaria (1-4) and asexually propagated (1)<br><br>Studies and examples of coral/Symbiodiniaceae separation (3)	DNA extraction sometimes troublesome (1)<br><br>Small polys mean acquisition strategy not easily controlled (2)<br><br>Brooding spawner (6,7,8)	Can be chemically rendered Symbiodiniaceae-free (6)?<br>Metabolomics profiles under ranging conditions already exist.
Stylophora	Geographically and environmentally widespread (1)	Brooding spawner (6,7)	Ease of coral/Symbiodiniaceae separation (5)?

	Mixotrophic. Acquisition strategy can be identified and controlled (3)<br><br>Easy to maintain in aquaria (1-4) and asexually propagated (1)<br><br>Can be chemically rendered Symbiodiniaceae-free (3,4)		Elemental profiles under ranging conditions exists.<br><br>Existing evidence of nutrient acquisition in different forms, dissolved, particulate and under thermal stress
